# Intestinal Dysbiosis Relating to Gut–Brain Axis and Behavior in Dogs: A Systematic Review with Text Mining Approach

**DOI:** 10.3390/ani16060986

**Published:** 2026-03-21

**Authors:** Arianna Del Treste, Luigi Sacchettino, Dario Costanza, Lucia Trapanese, Angela Salzano, Francesco Napolitano, Laura Cortese, Danila d’Angelo, Giuseppe Campanile, Adelaide Greco

**Affiliations:** 1 Department of Veterinary Medicine and Animal Production, University of Naples Federico II, 80137 Naples, Italy; arianna.del.treste@gmail.com (A.D.T.); luigi.sacchettino@unina.it (L.S.); angela.salzano@unina.it (A.S.); francesco.napolitano3@unina.it (F.N.); lcortese@unina.it (L.C.); danila.dangelo@unina.it (D.d.); giucampa@unina.it (G.C.); adelaide.greco@unina.it (A.G.); 2 Interdepartmental Center of Veterinary Radiology, University of Naples Federico II, 80137 Naples, Italy; 3 CEINGE-Biotecnologie Avanzate Franco Salvatore s.c. a r.l., 80145 Naples, Italy

**Keywords:** dog, intestinal dysbiosis, microbiota, microbiome-gut–brain axis, behavioral disorders, Text Mining

## Abstract

The intestinal microbiome plays a fundamental role in canine health and well-being, regulating functions, including digestion, immunity, metabolism, and behavior. Dysbiosis refers to the disruption of the balanced composition of resident commensal communities, and gut bacteria can influence behavior via neurological, metabolic, endocrine, and immune-mediated pathways. Growing evidence supports the existence of a bidirectional communication between the gut and the central nervous system, known as the gut–brain axis, through which intestinal microorganisms may influence behavior via neurological, metabolic, endocrine, and immune-mediated pathways. Despite the expanding interest in this field, the contribution of intestinal dysbiosis to the development and severity of behavioral and neurological disorders in companion dogs remains poorly understood. This review aims to critically analyze the literature from 2011 to 18 September 2025 concerning the association between dysbiosis, the gut–brain axis, and both gastrointestinal and non-gastrointestinal illnesses in dogs. To our knowledge, this review represents the first application of Text Mining (TM) in this domain: TM facilitates the identification and analysis of valuable information from extensive datasets, converting unstructured content into structured data, thereby enabling quantitative analysis. We used the following search terms on three bibliographic databases (PubMed, Scopus, and Web of Science): “dysbiosis” AND “canine” OR “dog” AND “gut–brain axis” AND “behavior”. Of the 1176 records retrieved, 35 studies were checked following the PRISMA guidelines, and they met the predefined inclusion criteria in the final analysis.

## Simple Summary

Dysbiosis, defined as an alteration of the physiological intestinal eubiosis, has been implicated in a wide range of pathological conditions, including behavioral disorders in dogs. Increasing evidence supports the existence of a bidirectional communication between the gut microbiota and the central nervous system in both humans and domestic animals, commonly referred to as the gut–brain axis. Despite growing scientific interest, the role of intestinal dysbiosis in modulating canine behavior remains incompletely understood. Therefore, this systematic review aims to comprehensively examine the association between intestinal dysbiosis, the gut–brain axis, and behavior in dogs by applying a Text Mining (TM) approach to the scientific literature published between 2011 and 18 September 2025.

## 1. Introduction

The canine intestine hosts a complex microbial ecosystem, named microbiota, comprising bacterial *phyla* such as *Actinobacteria*, *Bacteroidetes*, *Firmicutes*, *Fusobacteria,* and *Proteobacteria,* which support the host’s well-being and homeostasis [1]. The terms “eubiosis” and “dysbiosis” represent, respectively, a balanced microbial ecosystem and the disruption or loss of this equilibrium [1]. The concept of microbiome indicates not only microbial composition but also microbial structure, metabolites, and the surrounding environmental conditions [2].

In humans and dogs, changes in the intestinal microbiome and dysbiosis are associated with gastrointestinal diseases (e.g., chronic enteropathies, inflammatory bowel disease, etc.), pancreatic insufficiency, and other systemic disorders, such as cardiovascular disease and obesity [2]. A well-balanced microbiome exerts protective effects against inflammation, whereas chronic inflammation and persistent enteric dysbiosis are increasingly recognized as underlying mechanisms in multisystemic chronic conditions [2]. Acute diarrhea can also induce dysbiosis, leading to important alterations in fecal short-chain fatty acids (SCFAs), which are essential for communication between the brain and the gastrointestinal system [2].

Previous studies have also suggested that the intestinal microbiota is involved in various pathways (immune, endocrine, and neural) and that the intestine–brain axis can modulate not only behavior but also brain physiology [3]. In this context, inflammation has emerged as a potential contributor to behavioral and neurological disorders. Several studies have reported associations between intestinal dysbiosis and cognitive impairment, as well as the exacerbation of neurological conditions, such as epilepsy and behavioral disorders, including anxiety, in dogs [2,3,4,5,6,7]. These conditions may substantially impair the quality of life of both animals and owners and, in severe cases, contribute to abandonment or euthanasia [2,3,4,5,6,7]. On the other hand, evidence indicates that the transfer of fecal material from healthy donors to the gastrointestinal tracts of recipients with cognitive impairment improves their cognitive function, further supporting a causal link between gut microbiota composition and brain function [8].

With a bidirectional mechanism, the intestinal–brain–immune axis can modulate the gastrointestinal tract, and the gastrointestinal tract can influence the central nervous system’s activity [3]. Despite the studies mentioned above, the role of the microbiome in behavioral issues still needs to be thoroughly investigated [7] and this field of research remains immature, with inherent difficulties in comparing studies and limited opportunities for replication [9]. As a result, a comprehensive and systematic overview of how this topic has been addressed in the scientific literature is still lacking.

To address this gap, the present review applies a Text Mining (TM) approach to explore the existing literature and to support the analysis of the literature related to canine intestinal dysbiosis, the gut–brain axis, and behavior. TM is defined as a knowledge discovery process aimed at identifying and analyzing meaningful information within large volumes of textual data to reveal structures, patterns, associations, and emerging trends [10]. TM relies on machine learning (ML) techniques to transform unstructured textual content into structured data suitable for quantitative analysis, enabling the detection of semantic relationships between terms and thematic domains [11]. This exploratory and descriptive technique has already been applied in various areas, such as animal welfare, disease surveillance, and precision livestock farming [12,13,14]. To date, no TM analyses have specifically addressed the intersection of canine dysbiosis, the gut–brain axis, and behavior. In the present study, TM was incorporated as a complementary analytical layer alongside the conventional qualitative review. The primary aim of this work remains the integrative narrative synthesis of the available evidence, while the TM component was used to support thematic organization and to provide an objective overview of recurrent semantic patterns within the corpus. Rather than constituting the central endpoint of the study, TM functioned as an auxiliary tool to enhance transparency in structuring the discussion and to visually map conceptual relationships emerging from the literature. This combined approach allows the review to retain its qualitative depth while benefiting from an additional, structured perspective on how the field is organized and evolving.

## 2. Materials and Methods

### 2.1. Dataset

**Figure 1:**
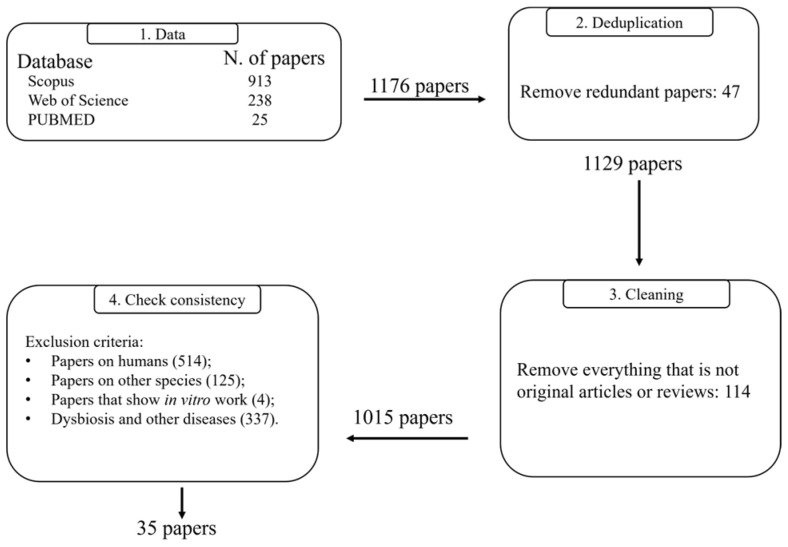
Flowchart of performed steps to obtain the final dataset.

A literature search protocol was established using three databases (PubMed, Web of Science, and Scopus) to identify peer-reviewed scientific papers addressing the possible relationship in dogs between the gut–brain axis and behavior, also relating to dysbiosis, using as search terms: ((((dysbiosis) AND (canine)) OR (dog)) AND (gut–brain-axis)) AND (behavior). The search, carried out in September 2025, was refined according to the publication period: 2011 to September 2025 (18 September, where possible to search with day/month/year, based on each database modality search). The research returned a total of 1176 documents coming from 913 (Scopus), 238 (Web of Science), and 25 (PubMed) documents, respectively. The retrieved records were compiled in an Excel workbook (Microsoft Excel^®^, v16.0), including for each paper the title, authors, affiliations, abstract, year of publication, document type (e.g., article or review), and source (journal or conference proceedings). Before analysis, a data-cleaning process was carried out, and the preprocessing workflow is summarized schematically in Figure 1. First, the duplicate records (47 papers) were eliminated since the same article could appear across multiple databases. Then, only journal articles and reviews were chosen, and hence, book chapters, proceedings, technical notes, and other types of documents were discarded (114 papers). At the end of the process, 1015 papers were analyzed by two independent authors (A.D.T and L.S), according to the PRISMA (Preferred Reporting Items for Systematic Reviews and Meta-Analyses) statement (Supplementary Materials: Figure S1) [15]. They provided a first independent selection and then double-checked. In case of disagreement, a third researcher (A.G) was consulted for a final judgment. Exclusion criteria were:

- Research in humans (514);
- Researchers regarding other species than dogs (125);
- Papers that show *in vitro* work (4);
- Dysbiosis and other diseases (337).

The screening process proved particularly challenging due to the marked heterogeneity of the literature initially retrieved (*n* = 1015 records). Although a large number of publications addressed intestinal dysbiosis in dogs, most did not investigate behavioral, neurological, or gut–brain axis-related outcomes or focused on systemic conditions unrelated to microbiota–behavior interactions. Additional complexity arose from the presence of studies conducted in other animal models, ex vivo experiments, pharmacokinetic analyses, or investigations linking dysbiosis to aging, environmental chemical exposures, or inflammatory diseases without direct behavioral relevance (dermatologic diseases, metabolic syndrome, obesity without behavioral endpoints, pancreatic disorders, cardiovascular disorders, and infectious enteropathies without CNS/behavioral endpoints). Only studies explicitly reporting behavioral, neurological, or gut–brain axis-related outcomes were included. As a result of these strict inclusion criteria, only 35 studies were ultimately deemed eligible for qualitative analysis. (Supplementary Materials: Table S1) The finalized dataset was then used to extract authors’ affiliations, publication year, journal, and country for profiling and descriptive analyses. The geographical affiliation of each paper was assigned based on the country of the corresponding author.

### 2.2. Text Mining (TM)

An unsupervised exploratory TM approach to identify dominant semantic domains and associations in a still immature and fragmented research field has been carried out. The abstracts of all selected papers were compiled into a separate Excel spreadsheet with two columns: *progressive ID* and *abstract*. This dataset was prepared for TM analysis. All subsequent operations were conducted in the RStudio environment 2020 [16] using functions from the packages *tm (version 0.7.11)*, *snowball (version 0.7.1)*, *ggplot2 (version 3.4.3)*, *dplyr (version 1.1.2)*, and *tidyverse (version 1.3.2)*. Following the preprocessing guidelines of Sebastiani 2002 [17], several preparatory steps were applied. Given the coexistence of American and British English, the corpus was standardized to American English. Preprocessing included tokenization, stop-word removal, and stemming/lemmatization. Tokenization segmented the text into meaningful units [18] by converting all words to lowercase, replacing escape symbols and special fonts (e.g., with white spaces), and removing punctuation, blanks, and numbers. Stop-word removal aimed to eliminate words irrelevant to semantic analysis—such as common articles, conjunctions, and terms already included in the search strings. The final preprocessing phase involved stemming and lemmatization [19] to reduce words to their root or dictionary forms. Stemming and lemmatization procedures applied during the TM workflow were necessary to standardize heterogeneous terminology across abstracts. It must be noted that these terms do not represent newly introduced variables but rather abbreviated or stemmed forms of expressions explicitly reported in the original studies.

While stemming may yield non-lexical roots, lemmatization returns valid dictionary words. To explore the textual content in depth, a Document–Term Matrix (DTM) was constructed, setting a three-letter minimum for inclusion, with documents as rows and terms as columns indicating term frequency [20]. The Term Frequency–Inverse Document Frequency (TF-IDF) method was then applied to weight each term according to its frequency within a document and its rarity across the corpus. This ensured that term importance reflected both frequency and uniqueness. Terms with TF-IDF ≥ 0.35 were used to create a histogram of the most frequent words. The TF-IDF threshold (≥0.35) was selected empirically to focus the visualization on terms with the greatest discriminative value, thereby facilitating the interpretation of thematic structures in the corpus. This parameter affects only the presentation of the co-occurrence networks and does not influence corpus inclusion. This procedure was in line with previous TM applications in the animal science literature [13,14]. Additionally, a word cloud was generated using the *wordcloud (version 2.6)* package, with word size proportional to the TF-IDF value, visually emphasizing the most recurrent terms. Finally, word associations were analyzed among terms with TF-IDF ≥ 0.35. Associations were measured based on co-occurrence frequency: values near 1 indicated frequent co-appearance, whereas values close to 0 suggested weak relationships. Only associations with coefficients ≥ 0.65 were reported in this study. A correlation coefficient threshold of ≥ 0.65 was adopted to identify strong semantic co-occurrence between terms, allowing the detection of robust thematic associations while preserving interpretability. These thresholds were chosen to balance sensitivity and specificity in an exploratory, unsupervised TM framework.

## 3. Results

### Descriptive Statistics

**Figure 2:**
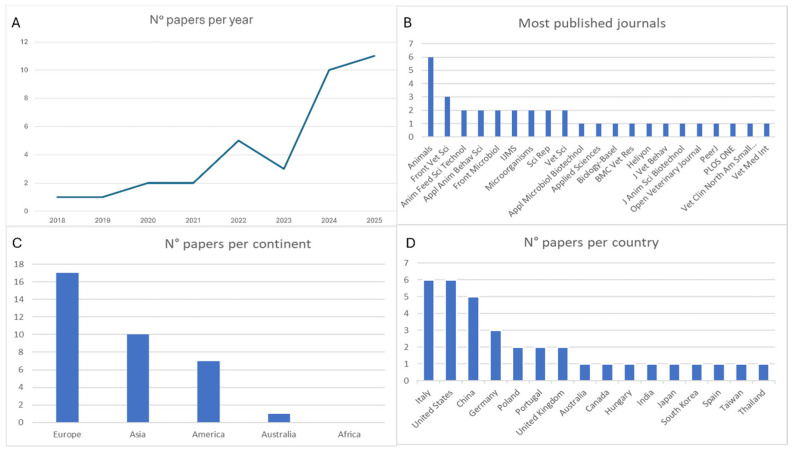
(**A**) Number of papers per year. (**B**) Number of papers for journals. (**C**) Number of publications per continent. (**D**) Number of publications per country.

The literature review identified a total of 35 peer-reviewed papers. In terms of publication type, research articles represented the majority, accounting for 62.9% (22/35) of the total records. They were followed by reviews (27.1%; 13/35). As illustrated in Figure 2A, the number of publications has increased. From 2018 to 2021, fewer than three papers were published annually. Starting in 2024, however, an upward trend seems to emerge, with a sharp rise in annual publications through 2025. Figure 2B presents the distribution of papers by journal, and the most prominent sources were Animals from MDPI and Frontiers in Veterinary Science, with six and three publications, respectively. Figure 2C,D shows the geographical distribution of the 35 papers based on the country affiliation of the corresponding authors. Europe emerged as the most productive continent, contributing 48.6% (17/35) of all publications, followed by Asia (28.6%; 10/35), North America (20%; 7/35), and Australia (2.8%; 1/35). At the national level, Italy and Germany were the leading European contributors (with six and three manuscripts each, respectively). In the Americas, the United States ranked first with six publications, while China led the Asian region with five papers. In Oceania, only one paper was produced by Australia.

**Figure 3:**
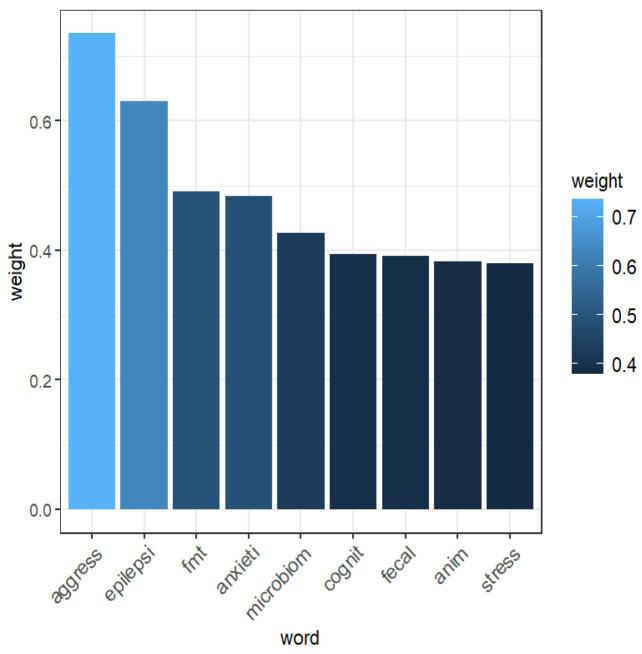
Histogram of the first 9 words (with a weight TF-IDF ≥ 0.35) extracted from 35 documents selected for inclusion in the study.

**Figure 4:**
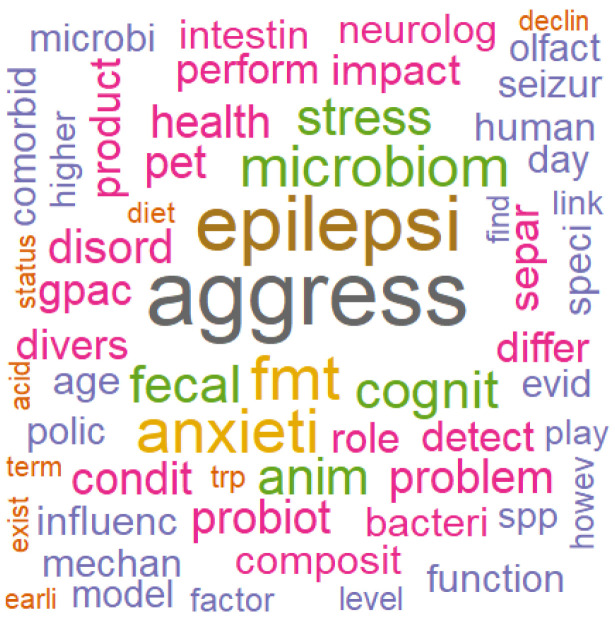
Word cloud with the most frequent words (TF-IDF ≥ 0.15).

After preprocessing and removing sparse terms (i.e., rare words), a total of 1205 terms were retained from the 35 abstracts analyzed. As shown in Figure 3, the most frequent terms with TF-IDF values ≥ 0.35 were identified. The highest-weighted terms included aggress (0.73), epilepsi (0.63), fmt (0.49), anxieti (0.48), microbiom (0.43), cognit (0.39), fecal (0.39), anim (0.38), and stress (0.38). A word cloud visualization (Figure 4) depicts these results, where the font size of each word is proportional to its TF-IDF weight, highlighting their relative importance within the corpus. Table 1 reports the word-association results, showing only terms with TF-IDF ≥ 0.35 and a correlation coefficient ≥ 0.65.

## 4. Discussion

To our knowledge, this is the first review addressing intestinal canine dysbiosis in relation to the gut–brain axis and behavior using a first exploratory TM approach. Traditional narrative and systematic reviews primarily aim to synthesize study design, methodological quality, effect size, and mechanistic evidence through qualitative or quantitative appraisal. In contrast, the TM-based approach adopted in the present study was intended as a complementary mapping tool rather than a substitute for critical evaluation. While conventional reviews allow in-depth interpretation of experimental protocols and biological mechanisms, TM provides a structured overview of thematic emphasis, semantic clustering, and research trends across the corpus. This approach reduces subjective narrative bias in thematic organization but does not quantify causal strength, consistency of evidence, or methodological robustness. Therefore, TM should be interpreted as a supportive analytical framework that enhances transparency in the literature structuring, while the core contribution of this review remains the integrative qualitative synthesis of the identified studies. TM has already proven effective in critically evaluating the scientific literature across different domains [12,13,14], allowing the identification of temporal trends and major thematic clusters. In the present review, TM was used to analyze the temporal evolution of the literature and to identify the main conceptual domains linking canine intestinal dysbiosis, gut–brain axis modulation, and behavior. To improve clarity and readability, the 35 selected papers were categorized according to their content, in line with the terms reported in Figure 3 and Table 1, which summarize the most relevant words (TF-IDF ≥ 0.35) and their semantic associations. The discussion is structured according to the dominant semantic domains emerging from the TM analysis. High-weight terms such as *aggression*, *epilepsy*, *stress*, and *FMT* identified through TF-IDF and co-occurrence analysis guided the thematic organization of the following sections, ensuring coherence between quantitative TM results and qualitative narrative interpretation.

### 4.1. The Intestinal Microbiota in Dogs

The normal canine gut microbiota is composed of several bacterial phyla, with *Firmicutes*, *Bacteroidetes*, *Fusobacteria*, *Actinobacteria,* and *Proteobacteria* being the most prevalent [1]. This complex microbial ecosystem plays a fundamental role in host homeostasis by regulating multiple physiological functions, including immune responses and metabolic processes [1,2].

Within this framework, our review highlights the importance of maintaining a balanced intestinal microbiota while also considering intestinal dysbiosis as a condition not limited to gastrointestinal disorders but also affecting behavior and brain disorders. Increasing evidence suggests that dysbiosis may influence distant organs and systems, particularly through the gut–brain axis, with potential consequences for canine behavior [9]. Accordingly, specific microbial metabolites, such as SCFAs, produced by bacterial fermentation of dietary fibers beyond providing energy to colonocytes, are involved in the modulation of inflammatory and immune responses and may influence cognition and neurotransmitter regulation [2,3,4,6].

### 4.2. The Intestinal Canine Dysbiosis: Symptoms and Influencing Factors

Canine dysbiosis could be related to poor diet, use of antibiotics and other medications, chronic stress, age, and underlying health conditions. Gut dysbiosis typically coincides with clinical manifestations indicative of compromised gut health, such as diarrhea. Diarrhea is the predominant symptom of chronic enteropathy in dogs, which constitutes the primary illness linked to alterations in the intestinal microbiota. The microbiota composition in dogs, particularly within the large intestine, predominantly consists of strict or facultative anaerobic bacteria; for example, secretory diarrhea is associated with a decrease in *Clostridium hiranosis* [5]. However, it varies individually according to age, diet, geographical location of the host, physical activity, breed, and gender [2,5]. Furthermore, clinical intestinal damage resulting from mucosal inflammation, as shown in age or diet-related dysbiosis, causes modifications in the microbiota and may lead to a decline in the dog’s physical activity [5]. The altered composition of gut microbiota has been associated not only with gastrointestinal disorders but also with various clinical conditions, including obesity, pancreatic and cardiovascular diseases, and inflammation-related disorders [2,5]. However, it was not the aim of this paper to describe these relations; we decided instead to focus only on the relation between dysbiosis and the brain axis, so we excluded numerous papers from the literature as described in the flow chart.

### 4.3. The Intestinal Microbiome: Assessment Techniques, Alterations, Possible Consequences, and Preventive Measures

Given the substantial inter-individual variability of the gut microbiome, no single gold-standard method is currently available to define or quantify dysbiosis [9]. Consequently, many approaches are employed to assess microbiome composition, including Polymerase Chain Reaction (PCR)-based algorithms, 16S rRNA gene sequencing, and fluorescence in situ hybridization (FISH) [5]. In addition to taxonomic profiling, the assessment of microbial metabolites—particularly SCFAs such as acetate, propionate, and butyrate—provides functional insights into gut health. Butyrate, in particular, represents a primary energy source for colonocytes and contributes to the maintenance of intestinal homeostasis and the reduction in inflammation [5]. SCFAs serve as a valuable energy source for mucus synthesis, intestinal epithelial cell regeneration, and the preservation of blood–brain barrier (BBB) integrity [5]. When investigating associations between gut microbiota and behavioral, gastrointestinal, neurological, or dermatological disorders, it is essential to consider both within-individual microbial richness and abundance (alpha diversity) and differences between individuals or groups (beta diversity) [21]. Despite growing evidence linking dysbiosis to various clinical conditions, knowledge regarding the specific impact of intestinal microbiota on canine behavior remains limited [2]. Canine intestinal dysbiosis can profoundly affect the overall health of the host. In humans, the ramifications of intestinal dysbiosis may be linked to certain psychiatric disorders [22]. In dogs, *Blautia* is among bacterial genera that significantly diminish during acute diarrhea, and its proportion has been linked to behavioral issues [7]. On the other hand, the intestinal microbes, such as *Lactobacillus* and *Bifidobacterium,* and their metabolites may influence canine behavior by participating in the synthesis of certain neurotransmitters and compounds like catecholamines [22]. Despite substantial research on canine intestinal dysbiosis concerning many clinical disorders, the understanding of the intestinal microbial impact on canine behavior remains restricted. From a preventive perspective, maintaining a balanced gut microbiome represents a key objective for canine health. Dietary optimization (tailored to age and physiological stage) and ensuring adequate protein, fiber, and carbohydrate intake are recommended as a primary strategy. Nutritional components and, in particular, high protein intake affect the dog’s microbiome; fat intake can affect microbial diversity and functionality. Soluble and insoluble fibers are considered the food of the good bacteria, necessary for fermentation and production of beneficial metabolites; the solubility and the complexity of carbohydrates also affect microbiota activity. The intestinal microbiota is responsive to nutrients, and a change in diet could affect the availability of substrates to gut microbes, leading to dysbiosis. A correct and controlled diet is crucial for maintaining the gut microbiota balance. Indeed, the equilibrium of the microbial community in the intestine not only pertains to gastrointestinal health but also affects the well-being of organs and systems physically remote from the gastrointestinal tract [1]. The administration of antibiotics can adversely impact the equilibrium and diversity of the gut microbiota; for instance, tylosin administration can alter the physiological composition of the canine intestinal microbiota, resulting in an increase in *Firmicutes* and a decrease in *Bacteroides*; furthermore, tylosin administration has been associated with an elevation in *Clostridium* and *Enterococcus* bacterial taxa [1]. Consequently, judicious and secure administration of antibiotics, in conjunction with synbiotic supplementation, can reduce the adverse effects of antibiotic therapy and rehabilitate the gut microbiome in dogs [1]. In our experience, it is advisable always to administer synbiotic supplements during or after antibiotic treatment to promote recovery of microbiome diversity, to restore the gut barrier function, and to relieve antibiotic-related gastrointestinal symptoms, also according to the most recent research papers [23].

### 4.4. Intestinal Microbiota and Future Therapeutic Perspectives

The World Health Organization (WHO) describes probiotics as live microorganisms that enhance health when given in specified quantities, and they are recognized for their benefits in animals, including both pets and livestock, in promoting gastrointestinal health [5]. Their use in companion animals has gained increasing attention due to their potential to support gastrointestinal and systemic health, although safety and strain-specific effects must be carefully considered; indigestible compounds known as prebiotics serve as substrates for beneficial intestinal microbes [24]. Synbiotic products, on the other hand, incorporate both prebiotics and probiotics, and postbiotics refer to formulations of inactivated bacteria [24]. Probiotics, prebiotics, and postbiotics all contribute to the equilibrium of the overall body’s homeostasis: probiotics are live beneficial microbes, prebiotics are nourishment for these microbes, and postbiotics are the beneficial compounds generated by probiotics. There are several studies [24,25,26,27,28] on the use of probiotics versus postbiotics in canine dysbiosis, but none of them are conclusive, and the research on the matter is still ongoing. Ongoing research into probiotics must prioritize the development of standardized protocols and guidelines to enhance their efficacy and safety [24]. Nutritional support associated with a multimodal therapeutic approach can assist specific behavioral and neurological issues (i.e., anxiety, epilepsy, and cognitive impairment) in dogs and cats [25]. Two studies have been conducted on *Lactobacillus*: a daily oral administration of probiotic strain *Lactiplantibacillus plantarum* PS128 for 14 days on dogs affects modulating the gut–brain axis, while another strain of *Lactiplantibacillus plantarum* (LP815^TM^) for 4 weeks ameliorated behavioral problems, suggesting a wider modulation on the gut–brain axis [26,27]. The administration of the yeast *Saccharomyces boulardii* in dogs indicated enhanced gut health and a significant decrease in fecal cortisol level, relating to chronic stress [28]. Additional nutraceutical approaches, including botanical compounds, polyphenols, essential oils, and complex supplements, have also been explored for their capacity to modulate the gut microbiota and influence behavior [29,30,31,32,33,34].

### 4.5. The Fecal Microbiota Transplantation (FMT)

The fecal microbiota transplantation (FMT) also represents a promising therapeutic strategy that entails the transfer of a healthy donor dog’s microbiota into dysbiotic dogs suffering from diarrhea, particularly those affected by infectious diseases and chronic enteropathies [5]. While additional investigations are required, FMT may prove to be highly beneficial in treating various conditions, including gastroenteric disorders (such as chronic enteropathies) and non-gastroenteric issues (like epilepsy and atopic dermatitis) [2,35]. FMT is also considered in our review as a possible treatment of intestinal dysbiosis: a randomized placebo-controlled study assessed the stress markers in police dogs correlated with microbiota alterations and their rectification using FMT for 14 days, which reduced diarrhea and improved serum stress profile and growth performance [36]. Despite the assessment of FMT treatment as a therapeutic tool for several canine somatic issues, studies investigating its potential effects on mitigating symptoms of behavioral canine disorders are limited [2]. A recent review pointed out the importance of gut microbiome modulation through FTM for Canine Cognitive Dysfunction (CCD), the dog version of human Alzheimer’s disease, revealing key insights and potential benefits for dogs and people with cognitive decline [8]. In this line, innovative strategies to counteract such a severe disorder rely on the gut–brain axis modulation, which makes use of non-conventional interventions like ketogenic diet, antibiotics, probiotics, and FMT, often associated with behavioral rehabilitation programs [6]. Watanangura et al. [37,38] also examined the beneficial effects of FMT on behavioral comorbidities in a canine model of epilepsy involving dogs with drug-resistant epilepsy and associated behavioral issues: the fecal donor experienced phenobarbital-responsive episodes without long-term seizures or notable behavioral changes. Results showed a lower abundance of both *Firmicutes* and *Blautia* and a higher abundance of *Ruminococcus*. An improvement of anxiety, fear, and Attention Deficit and Hyperactivity Disorder (ADHD)-like behavior, along with a reduction in excitatory neurotransmitters (aspartate and glutamate), an elevation in gamma-aminobutyric acid (GABA) levels, and the GABA/glutamate ratio, when compared to the baseline values [38].

### 4.6. Microbiota in Behavioral and Neurological Conditions in Dogs

A balanced gut microbiota is essential for endocrine, immune, metabolic, and neurological functions. Dysbiosis may contribute to systemic and central nervous system (CNS)-related disorders, including epilepsy, one of the most prevalent neurological conditions in dogs, affecting up to 0.75% of the dog population, with therapeutic options remaining limited, mainly due to cases resistant to available treatments [6]. Beyond pathological conditions, gut microbiota has also been implicated in aging and cognitive decline. Age-related microbial shifts have been correlated with memory performance in dogs, paralleling findings in human neurodegenerative disorders [22,39,40]. In particular, research highlighted a physiological link between aging, memory performance, and gut microbiota, as old dogs displayed lower levels of *Fusobacteria* and higher levels of *Actinobacteria*, which were associated with a higher number of mistakes on the short-term memory test [21]. A significant prevalence of some *Actinobacteria* was also documented in frozen and fixed post-mortem temporal cortex of human patients suffering from Alzheimer’s disease [39]. Moreover, in a very recent study [40] carried out in Beagle dogs, the authors showed that fecal SCFA concentration, especially acetate, increased with age, while propionate was higher in junior dogs, thus highlighting the importance of considering age-related factors and gut health biomarkers when investigating cognitive decline and CCD.

The possible correlation between the olfactory function of gut microbial communities in detection dogs, who are genetically predisposed to have excellent capabilities to sense different kinds of smells, has also been characterized [41]. A study analyzed the gut microbiota of German Shepherds, Labrador Retrievers, and Springer Spaniels, all housed in the same environment and fed the same diet, finding significant breed-dependent differences in their microbiomes [42]. Notably, German Shepherds showed greater richness and alpha diversity of the gut microbiome, with abundant *Prevotella*, *Alloprevotella,* and *Peptoclostridium*, thus emphasizing that high gut microbial diversity might correlate with better health, increased environmental resilience, and a more stable physiological state, potentially supporting enhanced olfactory functions. However, this data paves the way towards the targeted microbial manipulation to enhance sniffing performance in working canines [41].

The potential relationship between gut microbiota, bloodstream metabolites, and the expression of fear in dogs was recently explored [3]. In particular, fearful dogs were found to have a distinct gut microbiota composition, characterized by a significant increase in *Firmicutes*-related taxa, alongside lower levels of some *Proteobacteria*-related taxa, suggesting a putative mechanistic link between gut microbiota and emotional behavior via gut–brain axis pathways. In line with this, the correlation between gut microbiota composition and aggressive behavior is a highly researched aspect in dogs because of its significance for animal well-being and human safety. It has been seen that Pitbull dogs exhibiting conspecific aggressive behavior and housed at a temporary shelter under protective custody revealed higher *Firmicutes* and lower *Fusobacteria* and *Proteobacteria* when compared to non-aggressive breed-matched animals [43]. An interesting association between aggressive behavior and serotonin has been found, which may serve as a potential tool for identifying and monitoring aggressive tendencies in dogs [44,45]. According to what was previously found in dogs, as well as in humans and animal models, although significant differences in gut microbiome were not found in their experimental settings, aggressive working animals showed lower serum levels of 5-HT when compared to the non-aggressive groups, thus implying that the microbiome could aid in diagnosing and preventively intervening against aggression before its manifestation [2,45]. The gut microbiota in the enrolled subjects in a research paper showed that the predominant phyla *Bacteroidota*, *Firmicutes*, and *Fusobacteria* constituted approximately 95% of the gut microbiota, a result corroborated by other investigations on healthy dogs [7]. Moreover, the genus *Blautia* was consistently detected throughout investigations, indicating a correlation between this genus and anxiety in domestic dogs [7]. The gut microbiota and its impact upon aggressive and acute stress behaviors mainly stem from controlled experiments in mice and other animals [46,47,48].

### 4.7. Limitations

This study has some limitations that should be considered when interpreting the findings. First, the search strings may not have encompassed all possible synonyms or terminological variations related to canine dysbiosis, the gut–brain axis, and behavioral or neurological outcomes, potentially restricting the range of documents retrieved. In addition, the inclusion criteria, such as limiting records to those with abstracts available in English and applying predefined screening parameters, may have influenced the number and characteristics of the studies analyzed. Second, the TM approach was based primarily on abstracts rather than on a full-text evaluation of each article. While this strategy allows for the systematic processing of large volumes of the literature and facilitates the identification of thematic structures and term associations, it may not fully capture methodological nuances, effect sizes, or detailed mechanistic findings reported within the complete manuscripts. Despite these limitations, the study offers a structured overview of the thematic landscape characterizing research on canine gut dysbiosis and behavior, highlighting dominant research domains and areas where further integrative and mechanistic investigations are warranted.

## 5. Conclusions

In conclusion, intestinal canine dysbiosis and its relationships to the microbiome-gut–brain axis are a subject of great scientific interest that still needs to be investigated in relation to canine disorders and potential new treatments. We examined intestinal dysbiosis and how it contributes to the onset of brain illnesses, also considering multiple treatment strategies (i.e., use of prebiotics, probiotics, and fecal microbiota transplantation). Furthermore, TM allowed us to explore the relationships between canine dysbiosis and gut–brain axis in a systematic, yet discursive and easily comprehensible manner. The domain we evaluated necessitates continual updates and remains an underdeveloped area, requiring careful and scientifically rigorous contributions. While TM provides a structured and comprehensive overview, the dynamic nature of this research area implies the risk of missing recently published studies. Moreover, the fact that behavioral dysbiosis in dogs is still a little-understood field also poses a scientific challenge to be addressed and contributed to. In the future, both standardized research protocols and controlled studies on large canine populations are desirable to better understand the intestinal dysbiosis relating to the microbiome–gut–brain axis and behavior, as well as converting currently promising treatments into validated and clinically useful therapies.

## Figures and Tables

**Table 1:** Correlation for the most frequent words (TF-IDF ≥ 0.35) and the other words present in the documents analyzed. “cbarqbas” means the CBARQ baseline score, “fecal fatty” refers to fecal fatty acids, and “–h” refers to hours.

Word	Association (≥0.65)
aggress	earli (0.72), belgian (0.69), cbarqbas (0.69), china (0.69), clostridiumsensustricto (0.69), confirm (0.69), defens (0.69), diagnos (0.69), discriminatori (0.69), elisa (0.69), erysipelotrichaceaeucg (0.69), escherichiashigella (0.69), exploratori (0.69), facilit (0.69), femal (0.69), forest (0.69), human (0.69), lowest (0.69), male (0.69), malinoi (0.69), monitor (0.69), ngml (0.69), nonaggress (0.69), offens (0.69), phylumlevel (0.69), βdivers (0.69), strong (0.69), subgroup (0.69), subtyp (0.69), systemat (0.69), tool (0.69), year (0.69)
cognit	declin (0.92), similar (0.69), adassoci (0.69), Alzheimer (0.69), ccd (0.69), conduit (0.69), constant (0.69), correct (0.69), describe (0.69), dysbiosisassoci (0.69), goal (0.69), impair (0.69), materi (0.69), neurodegen (0.69), profound (0.69), reverd (0.69), rout (0.69), strike (0.69), version (0.69)
epilepsi	idiopath (0.90), innov (0.82), neurology (0.81), normal (0.80), appropri (0.75), convers (0.75), deepen (0.75), exacerb (0.75), fact (0.75), gutrel (0.75), preval (0.75), stand (0.75), stem (0.75), chronic (0.73), stabl (0.67), neural (0.66), signal (0.66)
fecal	acid (0.73), fatty (0.71), chain (0.68), immunoglobulin (0.66)
fmt	allevi (0.87), day (0.85), abstract (0.85), albumin (0.85), allobaculum (0.85), biochem (0.85), cetobacterium (0.85), cholesterol (0.85), cyan (0.85), diarrhea (0.85), elev (0.85), fece (0.85), gavag (0.85), glycerol (0.85), hour (0.85), kml (0.85), kunm (0.85), medium (0.85), microflora (0.85), modifi (0.85), point (0.85), polic (0.85), postfmt (0.85), road (0.85), salin (0.85), suspens (0.85), wolf (0.85), occur (0.82), transplant (0.81), low (0.76), transport (0.76), receiv (0.72), rate (0.70), growth (0.68), serum (0.68), dose (0.65)
microbiom	structur (0.73), accord (0.67)
stress	– h (0.82), stressed (0.82), acut (0.82), car (0.82), coloni (0.82), eightweek (0.82), event (0.82), expos (0.82), good (0.82), irrespect (0.82), meaning (0.82), predictor (0.82), previous (0.82), publish (0.82), react (0.82), repeat (0.82), timepoint (0.82), travel (0.82), whether (0.82), wider (0.82), twenti (0.76), fecal (0.66)

## Data Availability

No new data were created or analyzed in this study.

## Supplementary Materials

The following supporting information can be downloaded at https://www.mdpi.com/article/10.3390/ani16060986/s1. Figure S1: Flow diagram according to PRISMA statement; Table S1: Number of papers retrieved after preprocessing steps (*n* = 35).

## Abbreviations

TM	Text Mining
PRISMA	Preferred Reporting Items for Systematic Reviews and Meta-Analyses
SCFAs	Short-Chain Fatty Acids
ML	Machine Learning
DTM	Document–Term Matrix
TF-IDF	Term Frequency–Inverse Document Frequency
PCR	Polymerase Chain Reaction
16S rRNA	16S Ribosomal Ribonucleic Acid
FISH	Fluorescence In Situ Hybridization
BBB	Blood–Brain Barrier
WHO	World Health Organization
FMT	Fecal Microbiome Transplantation
CCD	Canine Cognitive Dysfunction
CNS	Central Nervous System
ADHD	Attention Deficit Hyperactivity Disorder
GABA	Gamma-aminobutyric Acid
5-HT	5-Hydroxytryptamine (serotonin)
