# Supplementary material for: Intestinal Dysbiosis Relating to Gut–Brain Axis and Behavior in Dogs: A Systematic Review with Text Mining Approach
Source: Animals (Basel). 2026 Mar 21;16(6):986. doi: 10.3390/ani16060986 (PMC13023595; doi:10.3390/ani16060986)
Supplement: Supplementary file 1 [file animals-16-00986-s001.zip › animals-4135334-supplementary.pdf]

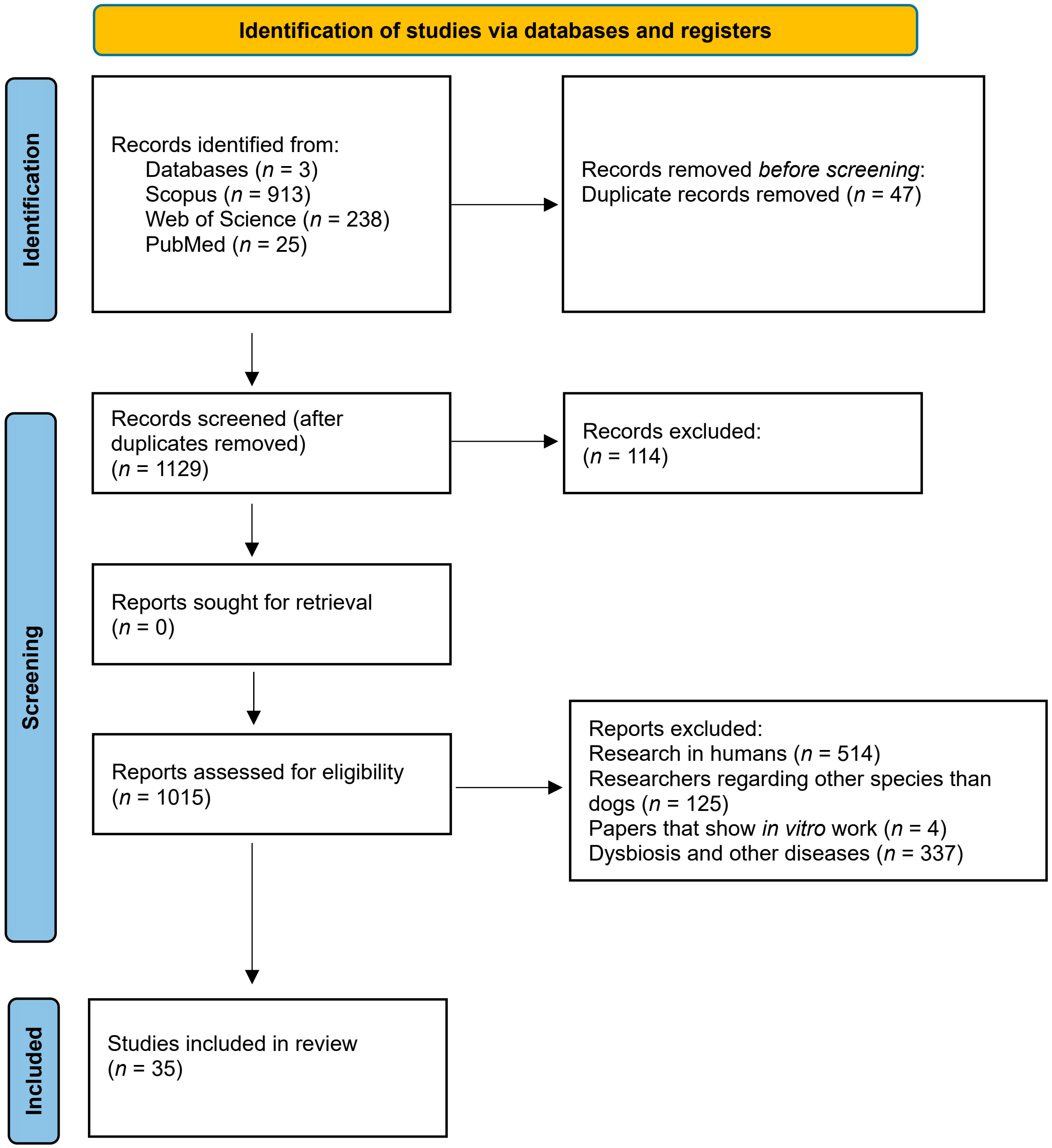

Figure S1: Flow diagram according to PRISMA

TITLE	FIRST AUTHOR	YEAR
A critical review of research concerning the gut microbiome in dogs and its relationship with behaviour	Crisante *et al.*	2025
A Diet Supplemented with Polyphenols, Prebiotics and Omega-3 Fatty Acids Modulates the Intestinal Microbiota and Improves the Profile of Metabolites Linked with Anxiety in Dogs	Ephraim *et al.*	2022
A six-month prospective, randomised, double-blinded, placebo-controlled, crossover, dietary trial design to investigate the potential of psychobiotics on seizure semiology and comorbidities in canine epilepsy: study protocol	Schmidt *et al.*	2023
Age-Related Changes in Gut Health and Behavioral Biomarkers in a Beagle Dog Population	Fernández-Pinteño *et al.*	2025
Altered microbiome and metabolome profiling in fearful companion dogs: An exploratory study	Sacchettino *et al.*	2025
Analysis and Comparison of Gut Microbiome in Young Detection Dogs	Li *et al.*	2022
Behavioral comorbidities treatment by fecal microbiota transplantation in canine epilepsy: a pilot study of a novel therapeutic approach	Watanangura *et al.*	2024
Composition, Bioactivities, Microbiome, Safety Concerns, and Impact of Essential Oils on the Health Status of Domestic Animals	Sivamaruthi *et al.*	2024
Decoding the Gut Microbiome in Companion Animals: Impacts and Innovations	Shah *et al.*	2024
Dietary grape proanthocyanidins modulate gut microbiome and neuroendocrine response in dogs	Sandri *et al.*	2024
Effect of a novel nutraceutical supplement (Relaxigen Pet dog) on the fecal microbiome and stress-related behaviors in dogs: A pilot study	Cannas *et al.*	2021
Effects of a Nutraceutical Treatment on the Intestinal Microbiota of Sled Dogs	Belà *et al.*	2024
Effects of fecal microbial transplantation on police performance and transportation stress in Kunming police dogs	Lin *et al.*	2024
Effects of Lactiplantibacillus plantarum PS128 on alleviating canine aggression and separation anxiety	Yeh *et al.*	2022
Effects of Saccharomyces boulardii Supplementation on Nutritional Status, Fecal Parameters, Microbiota, and Mycobiota in Breeding Adult Dogs	Meineri *et al.*	2022
Efficacy of a Novel Lactiplantibacillus plantarum Strain (LP815™) in Reducing Canine Aggression and Anxiety: A Randomized Placebo-Controlled Trial with Qualitative and Quantitative Assessment	Bijaoui & Zimmerman	2025
Exploring Gut Microbiota-Targeted Therapies for Canine Idiopathic Epilepsy	Blanquet *et al.*	2025
Exploring the Potential of Novel Animal-Origin Probiotics as Key Players in One Health: Opportunities and Challenges	Gorzelanna *et al.*	2025
Gut microbiome composition is associated with age and memory performance in pet dogs	Kubinyi *et al.*	2020
Gut microbiome structure and adrenocortical activity in dogs with aggressive and phobic behavioral disorders	Mondo et al.	2020
Gut Microbiota and Behavioural Issues in Production, Performance, and Companion Animals: A Systematic Review	Homer *et al.*	2023
Gut microbiota composition is related to anxiety and aggression scores in companion dogs	Pellowe *et al.*	2025
Gut-Brain Axis Impact on Canine Anxiety Disorders: New Challenges for Behavioral Veterinary Medicine	Sacoor *et al.*	2024
Impact of acute stress on the canine gut microbiota	Patel *et al.*	2024
Impact of Gut Microbiota on Host Aggression: Potential Applications for Therapeutic Interventions Early in Development	Mikami *et al.*	2023
Nutritional Management of Behavior and Brain Disorders in Dogs and Cats	Tynes & Landsberg	2021
Poop for thought: Can fecal microbiome transplantation improve cognitive function in aging dogs?	Dewey	2025
Resveratrol Ameliorates Chronic Stress in Kennel Dogs and Mice by Regulating Gut Microbiome and Metabolome Related to Tryptophan Metabolism	Bian *et al.*	2025
Study on the Correlation Between Aggressive Behavior and Gut Microbiota and Serum Serotonin (5-HT) in Working Dogs	Sun *et al.*	2025
The effect of phenobarbital treatment on behavioral comorbidities and on the composition and function of the fecal microbiome in dogs with idiopathic epilepsy	Watanangura *et al.*	2022
The gut microbiome correlates with conspecific aggression in a small population of rescued dogs (Canis familiaris)	Kirchoff *et al.*	2019
The microbiota: a key regulator of health, productivity, and reproductive success in mammals	Khan *et al.*	2024
The Relationship between Canine Behavioral Disorders and Gut Microbiome and Future Therapeutic Perspectives	Kiełbik & Witkowska-Piłaszewicz	2024
Understanding the diversity and roles of the canine gut microbiome	Kim *et al.*	2025
When the nose doesn't know: Canine olfactory function associated with health, management, and potential links to microbiota	Jenkins *et al.*	2018

Table S1. Number of papers retrieved after preprocessing steps (*n*=35).
